# Intra-tumoural microvessel density in human solid tumours

**DOI:** 10.1038/sj.bjc.6600315

**Published:** 2002-05-03

**Authors:** J Hasan, R Byers, G C Jayson

**Affiliations:** Cancer Research UK Department of Medical Oncology, Christie Hospital, Wilmslow Road, Withington, Manchester M20 4BX, UK; Department of Pathological Sciences, University of Manchester, Oxford Road, Manchester M13 9PT, UK

**Keywords:** microvessel density, angiogenesis, solid tumours

## Abstract

Over the last decade assessment of angiogenesis has emerged as a potentially useful biological prognostic and predictive factor in human solid tumours. With the development of highly specific endothelial markers that can be assessed in histological archival specimens, several quantitative studies have been performed in various solid tumours. The majority of published studies have shown a positive correlation between intra-tumoural microvessel density, a measure of tumour angiogenesis, and prognosis in solid tumours. A minority of studies have not demonstrated an association and this may be attributed to significant differences in the methodologies employed for sample selection, immunostaining techniques, vessel counting and statistical analysis, although a number of biological differences may account for the discrepancy. In this review we evaluate the quantification of angiogenesis by immunohistochemistry, the relationship between tumour vascularity and metastasis, and the clinicopathological studies correlating intra-tumoral microvessel density with prognosis and response to anti-cancer therapy. In view of the extensive nature of this retrospective body of data, comparative studies are needed to identify the optimum technique and endothelial antigens (activated or pan-endothelial antigens) but subsequently prospective studies that allocate treatment on the basis of microvessel density are required.

*British Journal of Cancer* (2002) **86**, 1566–1577. DOI: 10.1038/sj/bjc/6600315 www.bjcancer.com

© 2002 Cancer Research UK

## 

Angiogenesis is the process in which new blood vessels arise from existing ones. This is present physiologically in the adult during wound healing, the development of the ovarian follicle and corpus luteum and in the proliferating endometrium. Pathological angiogenesis is also a component of much diverse pathology ranging from diabetes and atherosclerosis to cancer, a disease that cannot progress without the formation of new blood vessels (Folkman, 1972).

The measurement of angiogenesis is complicated by the fact that it is a dynamic process yet most studies to date have focused on the product of angiogenesis, the microvessel density, which was analysed at a particular point in time. Accepting this caveat there are extensive data in which the microvessel density correlates with metastasis and survival. Here, we discuss the relationship between angiogenesis and the intra-tumoural microvessel density (IMD), review the different techniques that have been used to measure IMD and then discuss the reasons why this relationship does not always hold.

## IMD AS A MEASURE OF TUMOUR ANGIOGENESIS

As angiogenesis is a dynamic process, comparisons with snapshot views of the tumour, as are seen in biopsy analyses, are not straightforward. On the other hand the only approaches that are available in the clinic for the serial measurement of tumour angiogenesis involve molecular imaging strategies, such as contrast-enhanced magnetic resonance imaging (MRI) and positron emission tomography (PET), that do not resolve data at the microscopic level. Thus it is important to assess the relationship between IMD and angiogenesis.

Several studies have reported correlations between IMD and angiogenic growth factor expression, tumour growth and the occurrence of distant metastasis suggesting that IMD, as well as quantifying vascular density, reflects important information on the degree and function of tumour vasculature.

### IMD and angiogenic cytokine expression

Several clinicopathological studies have shown a direct association between angiogenic cytokine expression and IMD. Vascular endothelial growth factor (VEGF) expression has been shown to correlate with microvessel density in a number of solid tumours including prostate (Weidner *et al*, 1993), colon (Takahashi *et al*, 1995), lung (Mattern *et al*, 1996) and breast cancer (Linderholm *et al*, 1999). Toi *et al* (1995a) compared IMD with VEGF and PdEGF (platelet-derived endothelial cell growth factor) expression in 152 invasive breast cancer specimens. VEGF and PdEGF expression were significantly correlated with the increase in IMD, and in another study intra-tumoural VEGF concentrations in 135 breast cancer tissue homogenates were significantly higher in richly vascularised tumours as opposed to the ones that were poorly vascularised. (Toi *et al*, 1996). VEGF concentrations measured by ELISA in the tumour tissue from 19 brain tumour patients were significantly correlated with vascular density (Takano *et al*, 1996). Almost all tumour cells in the peripheral areas of brain tumours that contained a high amount of VEGF protein were associated with increased IMD on immunohistochemical staining.

Significant correlation between VEGF expression and IMD has also been reported for gastric (Maeda *et al*, 1996), endometrial (Giatromanolaki *et al*, 2001) and cervical cancer (Guidi *et al*, 1995).

The clinical importance of VEGF is emphasised by the fact that VEGF inhibition has been shown to significantly inhibit angiogenesis and tumour growth in *in vivo* models (Kim *et al*, 1993; Saleh *et al*, 1996). Consequently anti-VEGF agents are being developed as therapeutic strategies to inhibit tumour angiogenesis and progression. Clearly these cytokines act through specific signalling receptors and recent data have shown a relationship between cytokine concentration, signalling receptor and IMD. In a series of 121 endometrial carcinomas, VEGF expression was associated with increased angiogenesis and poor prognosis, but more importantly its expression was linked with an increased density of vessels expressing the KDR (kinase domain region) receptor at the invading tumour front. VEGF expression in the absence of VEGF/KDR-activated vasculature was a less important predictor of poorer survival, suggesting that the prognostic importance of VEGF is significantly improved when the combined VEGF/KDR status is assessed (Giatromanolaki *et al*, 2001).

A positive correlation between IMD and other angiogenic cytokines like basic fibroblast growth factor (bFGF) has also been reported (Li *et al*, 1994; Riedel *et al*, 2000; Sugamoto *et al*, 2001). However, several studies failed to find any correlation between bFGF and IMD and have questioned its relevance as an independent prognostic factor (Burian *et al*, 1999; Schmidt *et al*, 1999; Smith *et al*, 1999). It has been hypothesised that bFGF has a role only when it synergises with other growth factors like VEGF. Thymidine phosphorylase (TP or PDECGF), another important angiogenic factor, is an intracellular enzyme involved in pyrimidine metabolism, neural function and neovascularisation (Brown *et al*, 1998). One of its metabolites 2-deoxy-D-ribose is a potent mediator of angiogenesis (Folkman, 1996). The prognostic value of TP has been recognised in breast cancer (Fox *et al*, 1996, 1997a) and a correlation between TP expression and IMD has been reported for other solid tumours (Toi *et al*, 1995a; Matsuura *et al*, 1999; Ueda *et al*, 1999).

### IMD and uPA/PAI-1 levels

Background extracellular matrix proteolysis is one of the most important steps in angiogenesis. The urokinase-type plasminogen activator system (uPAS) consisting of urokinase plasminogen activator (uPA), an extracellular proteolytic enzyme produced by tumour cells, its receptor uPA-R and their corresponding inhibitors plasminogen activator inhibitors 1 and 2 (PAI-1 and PAI-2) are thought to play a major role in this process. uPA at the cell surface initiates a proteinase cascade leading to the breakdown of the extracellular matrix and thereby promoting cellular migration. The levels of uPA and its inhibitor PAI-1 are known prognostic factors in breast cancer. In a series of patients with breast cancer, uPA and PAI-1 contents were measured by ELISA in tissue extracts, in peripheral and central tumour tissue (Hildenbrand *et al*, 1995). uPA and PAI-1 levels were higher in the peripheral breast tumour regions, particularly in node-positive patients and there was a linear correlation between CD31^+^ IMD and uPA/PAI-1 levels.

Although these studies have demonstrated a correlation between particular angiogenic factors and IMD, there have been very few attempts to examine multiple angiogenic and anti-angiogenic factors in conjunction with IMD and clearly more work is needed in this area.

### IMD and intra-tumoural microvascular characteristics

It is of particular interest to know the degree to which the radiological assessment of tumour vasculature correlates with IMD. A number of techniques exist to assess this, including contrast enhanced magnetic resonance imaging (MRI) (Brasch *et al*, 2000; Brasch and Turetschek, 2000; Anderson *et al*, 2001) colour Doppler ultrasound (Huber *et al*, 1994; Delorme and Knopp, 1998; Cheng *et al*, 1999) and positron emission tomography (Fanelli *et al*, 1999; Anderson *et al*, 2001). There is some evidence to show that radiological imaging can detect differences in IMD. In one study slow and fast growing subtypes of a R3230 mammary carcinoma were implanted into mice and were subsequently analysed for both IMD by FactorVIII-related-antigen (FVIII-RA) and plasma volume. MR imaging-derived tumour plasma volume and permeability increased exponentially with increased capillary density. A more densely structured vasculature was found in the fast growing tumours and the increased vascular density correlated with estimates of plasma volume as assessed by MRI (Van Dijke *et al*, 1996). However, in human studies these correlations have not been so strong, for instance, in a recent study of cervical carcinoma there was no clear relationship between MRI and IMD (Cooper *et al*, 2000). On the other hand a study in glioma, where VEGF has been more strongly implicated in the tumour's pathogenesis, did identify a relationship between MRI findings and IMD (Tynninen *et al*, 1999).

## QUANTIFICATION OF IMD BY IMMUNOHISTOCHEMISTRY

Despite the fact that the majority of studies have identified IMD as an independent prognostic factor in solid tumours, several studies have questioned the finding. These discrepancies may be due to particular tumour biological factors that obscure the relationship but other issues such as staining methodology have also been implicated. In part the variation in the relationship between IMD and prognosis has arisen from a lack of standardised immunohistochemical techniques because of the wide range of antibodies, antigen retrieval methods, designation of high and low vessel count groups (cut-off points), patient groups, therapies and data (vessel quantification) interpretation. The correct identification of the vascular hot spot within the tumour and observer experience are two of the most important factors. In one study that compared the effects of different methodologies on estimates of tumour vascularity, archival specimens of breast, lung and oral carcinoma, oral dysplasia and normal breast tissue were investigated. Pretreatment of sections (enzymatic digestion, heating), endothelial markers (vWF-von Willebrand factor and CD31 antibodies), method of quantification (highest microvascular density, average microvascular density and microvascular volume) and inter-observer variations were all found to alter the estimated vascularity and interestingly the treatment of sections before staining was the variable that most significantly altered the calculated vascularity of tumours (Schor *et al*, 1998).

In order to overcome some of these problems an international consensus on the methodology and criteria for evaluation of IMD has been put forward (Vermeulen *et al*, 1996). The report proposes a standard method for IMD assessment and sets quality control standards aimed at improving reproducibility and inter-centre comparability with regard to the selection of representative tissue samples, tissue processing and immunostaining, selection of areas for microvessel enumeration and the technique of vessel counting within these areas. A training programme for the inexperienced pathologist is also recommended given the subjective methodology of vascular hot spot selection and identification of individual microvessels. Few comparative studies have evaluated the different methods of microvessel quantification – manual counting. Chalkley count and CIAS. Further prospective studies are needed to define the method of choice. A standard technique for the evaluation of IMD would facilitate comparison between different centres and enable the organisation of confirmatory multicentre trials on the prognostic and predictive value of IMD in human solid tumours.

### Endothelial cell specific antibodies

Intra-tumoural microvessels can be identified by immunostaining of endothelial cells. Two categories of human endothelial cell specific antibodies are currently available: the pan-endothelial cell markers and antibodies that bind selectively to activated or proliferating endothelium (Table 1

**Table 1:**
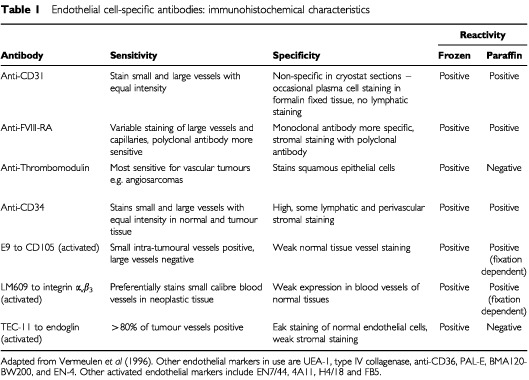
Endothelial cell-specific antibodies: immunohistochemical characteristics

). The pan-endothelial markers are characterised by equal intensity of staining for small and large vessels and reactivity in both frozen and paraffin embedded samples. The latter feature is of clinical importance in that it facilitates their use on archival specimens. Generally, CD31 is utilised as the pan-endothelial marker of choice for paraffin sections. In a recent review (Fox *et al*, 2001), when studies on breast cancer with multivariate analysis that used antibodies to CD31 or CD34 were examined, most revealed a positive association with relapse free survival (RFS) (13 out of 14) and all for overall survival (OS) (12 out of 12). With studies using FVIII-RA, only eight out of 13 were positive for RFS and seven out of 10 for OS.

The problem of antigen specificity is highlighted by the detection of CD34 antigen on lymphatic vessels, perivascular stromal cells as well as other stromal elements while this is compounded by the absence of FVIII-RA on part of the capillary endothelium in tumour tissue (Holthofer *et al*, 1982; Parums *et al*, 1990; Traweek *et al*, 1991; Miettinen *et al*, 1994). The disadvantages associated with staining for CD31 antigen include co-staining of inflammatory cells, but these can be distinguished from endothelial cells on the basis of morphological differences, and frequent antigen loss due to fixatives that contain acetic acid (Vermeulen *et al*, 1996). Microwave antigen retrieval effectively abolishes this problem but in prospective studies a careful selection of the most suitable tissue fixation procedure should still be performed.

The ability to distinguish quantitatively between tumour neovascularisation and pre-existing vessels may be important in the assessment of tumour angiogenesis and could provide more accurate prognostic information. This is now possible using markers for activated endothelium. These markers are suitable for proliferating endothelium giving none or poor staining of lymphatics and normal quiescent blood vessels. They mainly react with fresh or frozen tissues, their activity in paraffin-embedded specimens is fixation dependent. In a study of 106 patients with breast carcinoma, the IMD was assessed using a pan-endothelial marker CD34 and a monoclonal antibody to CD105 that preferentially reacts with endothelial cells in angiogenic tissues. IMD values for CD105 expression showed a statistically significant correlation with RFS (*P*=0.0362) and OS (*P*=0.0029) in contrast to blood vessel counts using CD34 that did not correlate with RFS or OS (Kumar *et al*, 1999). This discrepancy in findings between the two markers could be the result of variability in the reactivity of different endothelial cell antibodies. While anti-CD105 antibody specifically reacts with endothelial cells of blood vessels in tissues undergoing angiogenesis, anti-CD34 antibody generally binds to endothelial cells in large blood vessels, although its expression can be diminished or restricted in some tumour microvessels (Wang *et al*, 1994). The superiority of the anti-CD105 antibody over anti-CD34 was recently confirmed in another comparative study in 236 patients with NSCLC (Tanaka *et al*, 2001). CD105 determined IMD was a significant poor prognostic factor in multivariate analysis (*P*=0.029) while CD34 determined IMD was not.

The activity of another marker for activated endothelium; LM609, a monoclonal antibody against integrin α_v_β_3_ has been investigated in a cohort of primary breast cancers and in normal breast tissue as control. LM609 preferentially immunostained proliferating blood vessels of small calibre within neoplastic tissue with weak expression of the integrin observed on vessels of normal tissue. In a series of 197 breast cancers, the expression of integrin α_v_β_3_ at the vascular hot spot was the single most significant prognostic indicator for RFS in both node-negative and node-positive patients (Gasparini *et al*, 1998).

### The vascular hot spot

Vascular hot spots are regions of high vascular density within the tumour and were first defined in breast cancer (Weidner *et al*, 1991). It was hypothesised that vascular hot spots arise from angiogenic tumour cell clones and that these cells would predominantly enter the circulation and give rise to vascularised metastases. These areas are identified by an inspection of the tumour at low magnification. However, this is clearly time consuming when the diameter of some tumours often exceeds 10 cm. It was suggested that the number of vascular hot spots analysed should be at least 10 to reduce the chances of missing the most vascular areas (Martin *et al*, 1995).

From the practical standpoint, assessment of IMD in selected areas compared to an overall vascular count is less time consuming. Vascular hot spots are encountered predominantly at the peripheral tumour margin and can be selected by scanning a tumour section at low magnification (10–100×). A low background staining and highly specific and intense labelling of endothelial cells is required. Once the vascular hot spot is defined, a higher magnification is selected in order to be able to count individual microvessels. Magnifications of the order of 200–400×and field sizes ranging from 0.12 to 1.00 mm^2^ have been used (Vermeulen *et al*, 1996). A higher magnification improves the detail of the image and allows the identification of more single endothelial cell sprouts. An area larger or smaller than the vascular hot spot will result in loss of information.

According to an early study (Weidner *et al*, 1991), any highlighted endothelial cells or cell cluster clearly separate from adjacent microvessels, tumour cells and other connective tissue elements should be regarded as a distinct countable microvessel. This definition has several implications. Neither a lumen nor the presence of red blood cells is necessary to identify a microvessel. In addition a cut-off calibre size is not mentioned so that single cell sprouts as well as larger vessels are thus included in the counts. Even if distinct clusters give the impression of being part of one large vessel transfected by the plane of the tissue section more than once, they are counted as separate microvessels. Strict application of these objective criteria seems to result in lower inter-observer variability when analysing pre-defined hot spots.

Quantification of stained vessels can be achieved by measuring highest microvascular density (h-MVD), the average microvessel density (a-MVD) or the microvascular volume (MVV). The area of highest microvascular density (the vascular hot spot) is located by scanning the section at 100×magnification. In practice, localisation of the highest density area normally involves counting up to three different areas. Three different fields are counted in each of these areas at 200×magnification, and the highest value taken as the h-MVD, expressed as vessels per mm^2^. The a-MVD is determined using the same grid and magnification (200×) as for h-MVD and calculating the mean of the vascular counts obtained in at least 10–15 random fields for each tissue section. Results for a-MVD are expressed as mean±standard deviation (vessels per mm^2^) (Schor *et al*, 1998). The MVV is estimated by point counting using an eyepiece graticule, which contains 100 points. Vessels that coincide with the points are counted in 15 fields selected randomly across each section (a total of 1500 points) and yields results expressed as percentage volume.

The Chalkley method resembles that used to determine the MVV. Tumour sections are scanned at low magnification to identify the areas that appear to have the maximum number of discrete microvessels. At higher magnifications, an eyepiece graticule containing 25 randomly positioned dots is rotated so that the maximum numbers of points are on or within the vessels of the vascular hot spot. Instead of counting the individual microvessels, the overlying dots are counted. In a series of patients with breast carcinoma (Fox *et al*, 1995), a significant correlation was found between MVD assessment by the Weidner method and Chalkley point counting (*r*=0.7, *P*=0.00005). A significant reduction in OS was observed between patients stratified by Chalkley count in both univariate (*P*=0.02) and multivariate analysis (*P*=0.05). In another study in patients with node-positive breast carcinoma, Chalkley score was found to be the most significant independent predictor of outcome by multivariate analysis (Gasparini *et al*, 1996). In a series of 330 breast cancers (Rose *et al*, 2000), Chalkley count was compared to manual microvessel counting and provided independent prognostic value in multivariate analysis (*P*<0.0001 for RFS, *P*=0.001 for OS). Manual microvessel counting had no prognostic impact. In another study of 104 malignant mesotheliomas, IMD as assessed by Chalkley counting was assessed with respect to other known prognostic factors in malignant mesothelioma. Chalkley microvessel count was shown to be an independent prognostic factor in multivariate analysis (*P*=0.006) (Edwards *et al*, 2001). Since no decisions have to be made on whether adjacent stained structures are stained microvessels or not, Chalkley point counting should be a more objective approach.

The first problem that arises with the quantification techniques is the selection of a representative tumour block. In colorectal adenocarcinoma, the IMD in in-situ growth regions is approximately half of that seen in invasive regions (Vermeulen *et al*, 1995) suggesting that multiple blocks should be assessed. de Jong *et al* (1995) found a higher average coefficient of variation (24%) if more than one tissue block was analysed compared to a lower coefficient of variation (15%) when only counts within sections of one block were examined, indicating that a comprehensive inspection of available tumour material is needed to identify the relevant hot spots.

The training and experience of the investigator influences the identification of the vascular hot spot. Barbareschi *et al* (1995) compared the calculated IMD in 91 node negative invasive breast carcinomas by light microscopy when measured by two pathologists of different experience. Both at univariate and multivariate analysis, only the counts of the experienced pathologist were significantly associated with relapse-free survival. Similar results were noted in another series of node negative breast cancer patients (Vermeulen *et al*, 1997). Once the vascular hot spot is identified, vessel counts after agreement on the description of a single countable vessel appears to be less dependent on subjective interpretation than the process of hot spot selection.

### Grading of IMD

In addition to the vascular hot spot technique another method is semiquantitative grading. Several studies have reported a positive correlation between quantitative and semiquantitative MVD scores. Weidner *et al* (1991) subjectively graded angiogenesis in vascular hot spots and counted individual microvessels in the same fields (Weidner *et al*, 1992). MVD values obtained by both methods were a statistically significant predictor of RFS and OS. The obvious advantage of IMD grading is its time efficiency. However, translation of a continuum of MVD values into a categorical type of data will however be associated with some loss of information. Given the highly subjective nature of IMD grading, comparable results will only be obtained by different observers after a period of training with standardised methods.

### Computerised Image Analysis systems (CIAS)

This is an automated counting technique that improves reproducibility and reduces inter-observer variability and has been proposed as a more objective method of assessing IMD (Wakui *et al*, 1992; Visscher *et al*, 1993). In a series of 91 node-negative invasive ductal carcinomas of the breast, both the number of CD31 positive microvessels measured by an experienced observer and the microvessel area (MVA) determined by CIAS were independently associated with RFS (Barbareschi *et al*, 1995). Fox *et al* (1995) also reported comparable results in another series of patients with breast carcinoma.

The main advantage of CIAS is the additional morphometric parameters that can be detected i.e. the number of vessels with a certain dimension range, the vessel luminal area, vessel luminal perimeter and the number of immunostained areas per microscopic field. IMD can be measured more objectively without the intervention of an investigator. The apparent disadvantage of CIAS is the time consuming nature of the method and its higher cost. These systems are not fully automated yet and require a high degree of operator interaction. The vascular hot spot is still identified manually before automated counting as the heterogeneity of microvessel morphology and immunostaining intensity particularly hampers a fully automated analysis of tumour IMD.

## IMD AND METASTASIS

As tumour metastasis is the major cause of mortality in cancer patients, a classification of the metastatic potential of a tumour could be of great clinical significance. A quantitative relationship between IMD, the number of intravascular tumour cells and the occurrence of pulmonary metastasis was shown in an animal tumour model more than 20 years ago (Liotta *et al*, 1974) but clinical confirmation has only been revealed in the last few years (McCulloch *et al*, 1995). Before surgery for patients with primary breast cancer, a central venous catheter was fixed in the ipsilateral subclavian vein. Blood samples were taken before, during and 1 day after surgery and tumour cells were identified on the basis of cytokeratin positivity. Microvessels were highlighted with anti-CD34 antibody in tissue sections of the invasive tumour component and the IMD was measured in the vascular hot spots. The majority of patients with a high vascular density had cells detected during operation in contrast to a minority of patients with a low IMD, suggesting that the frequency of tumour cell shedding was related to IMD (*r*=0.56, *P*=0.024).

Evidence that the intensity of angiogenesis in a human tumour could predict the probability of metastasis was initially reported in cutaneous melanoma (Srivastava *et al*, 1986, 1988). There was a clear distinction between a stage without neovascularisation, which correlated with a low rate of metastasis, and a stage in which increasing neovascularisation correlated with an increased rate of local and distant metastasis (*P*=0.025). Weidner *et al* (1991), in one of the earliest studies, reported a significant correlation between the degrees of IMD at the vascular hot spot with the probability of metastasis in a series of 49 patients with invasive breast cancer. Using light microscopy, blood vessels were highlighted by staining their endothelial cells immunocytochemically for factor VIII. The microvessels were counted (per 200×field), and their density graded (1–4+) in the most active areas of neovascularisation. Both microvessel counts and density grades were found to correlate with metastatic disease. For each 10-microvessel increase in the vessel count per 200×field, there was a 1.17-fold increase in the risk of distant metastasis (*P*=0.029). In another series of patients with node-negative breast cancer, assessment of IMD by immunohistochemistry with anti-CD31 antibody, IMD correlated significantly with recurrence in viscera, bone and soft tissue deposits (Gasparini *et al*, 1994). Several studies have now shown that highly vascularised tumours have a significantly higher likelihood to present with loco-regional lymph node metastasis than those that are poorly vascularised. Likewise a similar correlation between IMD in the primary tumour and the development of distant metastasis was found (Table 2

**Table 2:**
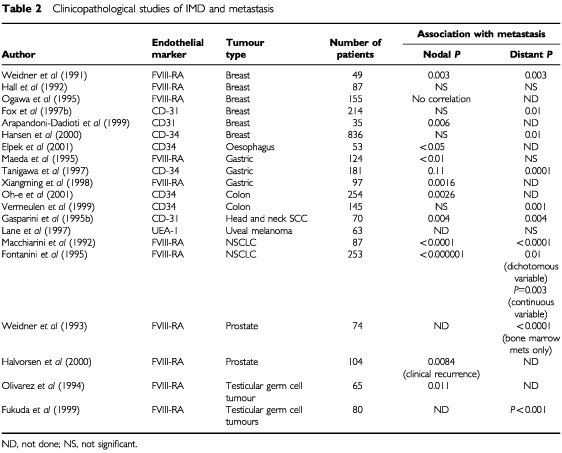
Clinicopathological studies of IMD and metastasis

).

The expression levels of genes that regulate metastasis and angiogenesis can predict metastatic potential in individual patients. This was demonstrated in a study of 46 renal cancers (Slaton *et al*, 2001). The expression levels of basic fibroblast growth factor, vascular endothelial growth factor, interleukin-8 (IL-8), matrix metalloproteinases (MMP) 2 and 9 and E-cadherin were examined at the periphery of the tumour by a colorimetric *in situ* mRNA. The expression levels of bFGF, VEGF, IL-8, MMP 2 and 9 were significantly higher in primary renal tumours from patients with synchronous or metachronous metastases than those who were disease free at a median of 48 months of follow up. Multivariate analysis of RFS showed that the ratio of MMP-9 to E-Cadherin (*P*=0.012) and the expression level of bFGF expression (*P*=0.045) were independent predictors for the development of metastases.

The expectation that determination of an angiogenic index by IMD can identify all patients with occult metastatic disease or those with probable distant metastasis is probably unrealistic. Human tumours are heterogeneous and consist of subpopulations of cells with different biological properties. Secondly, the process of development of metastasis consists of a series of interlinked independent steps. To produce clinically relevant metastasis, tumour cells must complete all the steps in this process. Tumour cells that can induce intense angiogenesis but cannot survive in the circulation or proliferate in distant organs will not produce metastasis (Ellis and Fidler, 1996). Like the other steps in the metastatic cascade, angiogenesis is necessary but not sufficient for the pathogenesis of metastasis.

## IMD AS A PREDICTIVE MARKER OF RESPONSE TO ANTI-CANCER THERAPY

Although IMD has been shown to correlate with metastasis and survival one might anticipate that the increased vascularity may improve tumour oxygenation and drug delivery thereby improving the response to therapy. However, the converse has generally been reported (Table 3

**Table 3:**
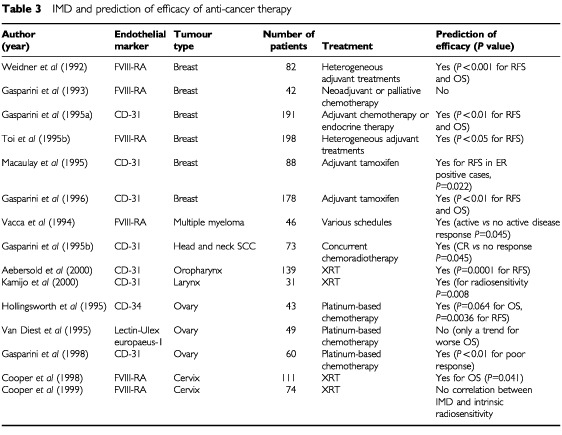
IMD and prediction of efficacy of anti-cancer therapy

). In a study of patients with squamous cell carcinoma of the head and neck treated with concurrent chemoradiation, the degree of IMD was significantly predictive of poor response to platinum-based chemotherapy in terms of complete response (*P*=0.045) (Gasparini *et al*, 1995b). Similar findings have been observed in patients with epithelial ovarian cancers treated with platinum-based combination chemotherapy (Hollingsworth *et al*, 1995).

If patients present with highly angiogenic primary tumours and these are the patients most likely to develop distant recurrences, then it might follow that this group of patients are most likely to benefit from adjuvant therapy. However in contrast to this hypothesis, the above studies demonstrate that angiogenic tumours have a more aggressive phenotype and do not benefit as anticipated. Perhaps tumours of low angiogenic index are more likely to benefit from adjuvant therapy even though they are also the subgroup of patients least likely to develop a recurrence. Perhaps patients with highly angiogenic tumours should therefore be selected for trials that include the use of anti-angiogenic strategies. The general observation from the above data is thus that the greater the degree of vascularisation in a solid tumour, the lower the likelihood of responsiveness to conventional anti-cancer therapy although this conclusion needs further evaluation in well-designed prospective studies.

## IMD AND PROGNOSIS

If IMD is associated with metastasis then it is logical to predict that IMD will also be associated with survival and this has been evaluated in most solid tumours including breast, ovary, bladder, head and neck and prostate cancers amongst others (Tables 4

**Table 4:**
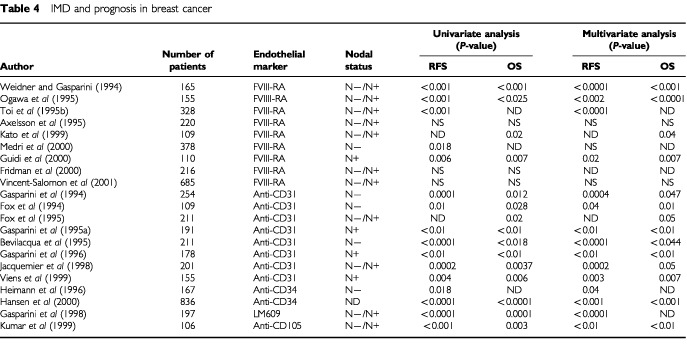
IMD and prognosis in breast cancer

and 5

**Table 5:**
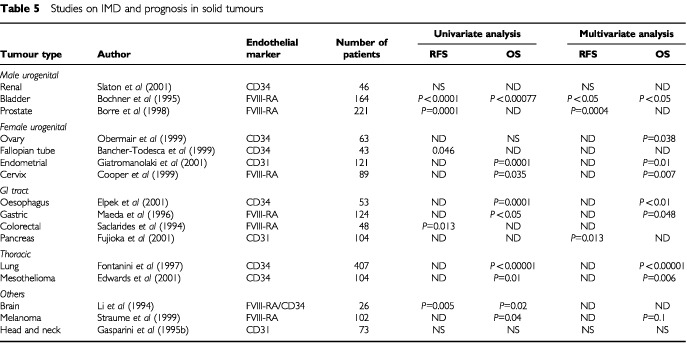
Studies on IMD and prognosis in solid tumours

). Two pivotal independent studies by Horak *et al* (1992) and Weidner *et al* (1992) found that IMD is a significant and independent prognostic factor in human invasive breast cancer. In a prospective blinded study of 165 patients with breast cancer, Weidner and Folkman showed that there was a highly significant association between IMD and RFS and OS in both node-negative and node-positive subsets (*P*=0.01). All patients with breast carcinomas containing more than 100 microvessels per 200×field experienced tumour recurrence within 33 months of diagnosis, compared with less than 5% of patients with breast cancer having 33 or fewer microvessels per 200×field. Moreover IMD was the only statistically significant predictor of OS in node-negative patients (*P*<0.001). In the study by Horak *et al* (1992) microvessels were counted in 103 patients with primary breast cancer using the JC70 antibody to CD31. Tumours showed significantly higher vascularisation than normal breast tissue and the number of blood vessels/mm^2^ was significantly associated with node metastasis (*P*<0.00001). Vascularity was correlated with the size of the primary tumour and with poor differentiation. Within each subgroup of size or differentiation, tumours without nodal involvement had much lower IMD. Even with the short follow up in this study, high vascular counts correlated with early deaths.

Over a hundred retrospective studies have been published on the prognostic significance of IMD in solid tumours. In breast cancer alone more than 40 retrospective studies have been published with more than 7000 patients evaluated. Since these patients have different pathological and clinical characteristics, a proper metaanalysis is not possible. Nevertheless more than 75% of studies reported positive results on the association of IMD with clinical outcome and more than 85% of the 27 studies, which included multivariate analysis, found that IMD was an independent prognostic variable (Gasparini and Harris, 1995; Ellis *et al*, 1998; Gasparini, 2001) and these findings have been observed in both the adjuvant and metastatic disease settings (Gasparini *et al*, 1995a, 1996; Macaulay *et al*, 1995; Toi *et al*, 1995b). The bulk of accumulating data indicates that IMD in the area of most intense neovascularisation in invasive breast cancer is an independent, significant and accurate prognostic marker in predicting poorer survival. Such an indicator might be potentially useful in tailoring adjuvant therapy for high-risk breast cancer patients although there is no data at present linking aggressive treatment to better outcome in such patients. Clearly prospective evaluation of this area is required.

The prognostic value of IMD has been studied in other solid tumours apart from breast cancer. These include cancers of the lung, genito-urinary tract, GI tract, head and neck, gynaecological malignancies and malignant melanomas. In the largest single series, IMD was assessed prospectively in 407 patients with stages I–III non-small cell lung cancer (NSCLC). Anti-CD34 antibody was used to measure angiogenesis in tumour samples. In multivariate analysis IMD, tumour microvessel count (*P*<0.00001), tumour size and regional lymph node status retained independent prognostic value with respect to overall survival. Among these variables, tumour microvessel count considered as a continuous variable was the most important (Fontanini *et al*, 1997).

### Poor associations between IMD and survival

There are several reports that do not demonstrate a relationship between IMD and survival (Hall *et al*, 1992; Van Hoef *et al*, 1993; Axelsson *et al*, 1995; Costello *et al*, 1995; Goulding *et al*, 1995; Morphopoulos *et al*, 1996). While these studies may reflect a genuine biological finding there are a number of technical issues that may confound studies. For instance a discrepancy may be related to potential methodological pitfalls in case selection, small study populations, heterogeneous therapy, inadequate follow-up and statistical analysis. Issues related to the application of immunohistochemical techniques including choice of endothelial marker, the vascular variable quantified and the area of tumour section assessed and inter-observer variability could also contribute. On the other hand, the tendency for positive results to be published may have led to an exaggerated assessment of the importance of IMD.

It is possible that some tumours are less angiogenesis-dependent than others. For example, in intestinal-type gastric cancer, vessel counts correlate with stage of disease and metastasis formation (Takahashi *et al*, 1996). In contrast vessel counts in diffuse-type gastric cancer do not correlate with metastasis and in general vessel counts in diffuse-type gastric cancer are lower those seen in intestinal-type gastric cancer.

Tumours may also be able to grow without neovascularisation if a suitable vascular bed is available. The pattern of vascularisation was studied in 500 cases of non-small cell lung cancer. Of these 80 (16%) were characterised by an absence of stroma and lack of new vessel formation (Pezzella *et al*, 1997). In such cancers, the degree of neovascularisation as assessed by IMD may not be of prognostic value. On the other hand, in colon cancer, the presence of ulceration and adjacent inflammation may itself contribute to increased local vascularity independent of the tumour and may confound results (Abdalla *et al*, 1999). Discordant results from studies may also reflect the fact that angiogenesis is but one step in the multistep process of metastasis. If a primary tumour has a high angiogenic index, but does not express other factors necessary for metastasis formation (i.e. adhesion/cohesion molecules, motility factors, growth factor receptors, etc.), then despite the high degree of angiogenesis, metastasis will not occur.

A further mechanism by which the discrepancy between IMD and survival may occur is vasculogenic mimicry, which has been described in melanoma. In aggressive primary and metastatic melanomas, the tumour cells generate microcirculatory channels composed of extracellular matrix that are lined by tumour cells (Maniotis *et al*, 1999; Folberg *et al*, 2000). The channels generated through this process by tumour cells may not stain with endothelial cell markers, as endothelial cells are not present.

Despite the fact that the overall trend in most studies is that the assessment of IMD retains a prognostic value in the majority of solid tumours, no definitive conclusions can be drawn at present on the real clinical usefulness of this approach. Before adopting IMD as a prognostic marker in routine clinical application, appropriate prospective trials are needed to validate the results observed in retrospective studies. Furthermore the method of assessment of IMD is inconsistent and needs to be standardised and made more objective.

## CONCLUSION

There is sufficient evidence to justify the determination of IMD as a measure of the angiogenic activity of human solid tumours. This method may be improved by employing more selective and specific markers for activated/proliferating endothelium, improving staining techniques, using more objective and reproducible methods for microvessel counting and standardisation of the criteria for evaluation of IMD and of the identification of the neovascular hot spot within each tumour.

As alternative methods like serum and tissue sampling of angiogenic peptides, measurement of endothelial cell proliferation, cell adhesion molecules and proteolytic enzymes as well as dynamic contrast enhanced MRI become available, these may enhance the accuracy of the measurement of angiogenic activity in solid tumours.

A strong correlation has been confirmed between the IMD of a primary tumour and its potential to develop locoregional or distant metastasis. Assessment of tumour vascularity is now an established prognostic indicator in early stage breast cancer, NSCLC and prostatic carcinoma (Gasparini and Harris, 1999). In these tumour types, the majority of retrospective studies found a significant correlation between vascularisation of the primary tumour and clinical outcome of patients. In other tumour types the role of IMD remains investigational and requires well-designed prospective studies. Preliminary studies suggest that the determination of angiogenesis may serve as a marker to predict response to some forms of conventional anti-cancer therapy. An inverse relationship between the degree of vascularisation and responsiveness to anti-cancer therapy has been shown in the adjuvant setting as well as for advanced tumours.

Certain issues remain unresolved. Besides the technical standardisation issues, it is unclear whether antibodies directed against activated endothelium will predict metastasis and survival better than other pan-endothelial methods. This issue needs to be addressed in a large study before we can establish prospective trials that are ultimately needed to validate IMD as a guide to therapy.
